# Women with lipoedema: a national survey on their health, health-related quality of life, and sense of coherence

**DOI:** 10.1186/s12905-022-02022-3

**Published:** 2022-11-18

**Authors:** Johanna Falck, Bo Rolander, Annette Nygårdh, Lise-Lotte Jonasson, Jan Mårtensson

**Affiliations:** 1 Department of Nursing Science, School of Health and Welfare, Jönköping University, Jönköping, Sweden; 2 Futurum, Academy for Health and Care, Jönköping County Council, Jönköping, Sweden; 3 Department of Social Work, School of Health and Welfare, Jönköping University, Jönköping, Sweden

**Keywords:** Comorbidity, Health, Lipoedema, Quality of life, Sense of Coherence, Surveys and Questionnaires, Women’s Health

## Abstract

**Background:**

Lipoedema is a chronic disease in adipose tissue that almost exclusively affects women during periods of hormonal alterations. Its main symptoms include an abnormal accumulation of subcutaneous fat in the buttock, hips, and legs, which is associated with pain, swelling, and easy bruising. Herein, a grading in three stages is used to determine disease progression. Problematically, lipoedema manifestations are often confused with lifestyle-induced obesity, which is why the various health problems among affected women often remain unrecognized. Overall, research on lipoedema is scarce. As such, this study examined the health, health-related quality of life (HRQOL), and sense of coherence (SOC) among women with lipoedema.

**Methods:**

We conducted a national cross-sectional study using an online survey assessing sociodemographic data, lipoedema characteristics, symptom severity, comorbidities, HRQOL (RAND-36), and SOC (SOC-13). In total, 245 women with lipoedema, recruited from all Lipoedema Association groups in Sweden, participated. Data were compiled with descriptive statistics, and mean differences between groups were analysed by using parametric and non-parametric tests.

**Results:**

Moderate and severe leg heaviness, pain, numbness, cold skin, feeling cold, easy bruising, and sleep problems were found to occur in all lipoedema stages. Moreover, almost all participants reported having comorbidities. Worse physical health and most substantial limitations in daily life were reported among women with the most progressive lipoedema (i.e., stage 3). Social and emotional functioning and SOC were found to be, on the other hand, primarily related to respondents’ sociodemographic data and their ages at lipoedema onset. Even though approximately 70% of the women had experienced lipoedema onset before age 30, only three (1.6%) had been diagnosed by a healthcare professional before that age.

**Conclusion:**

Having lipoedema is associated with several health problems and a lower HRQOL. In addition, the extent of delay in diagnosis within this sample indicates that many women with lipoedema are often underdiagnosed and are left without support from healthcare. These findings call for the need for greater attention on lipoedema. Moreover, further studies on how women with lipoedema manage their health and symptoms, as well as on their experiences of healthcare services and lipoedema treatments, are needed.

**Supplementary Information:**

The online version contains supplementary material available at 10.1186/s12905-022-02022-3.

## Background

Lipoedema is a chronic and progressive adipose disease involving loose connective tissue that almost exclusively affects women. The disease manifests as an abnormal and symmetrical accumulation of nodular and fibrotic subcutaneous fat, mainly in the lower extremities, and which is associated with pain, swelling, tenderness on pressure, and easy bruising in the affected areas [1]. Although lipoedema was described as far back as the 1940s by Allen and Hines [2], it is still considered as being relatively unknown in healthcare and is often misdiagnosed as lifestyle-induced obesity or lymphoedema [3]. Consequently, available data on its prevalence are sparse and divergent. However, previous studies have revealed that lipoedema may affect approximately 10% of the female population [4–6].

The etiology and pathophysiology of lipoedema are not fully understood. However, feminine hormones (estrogen) appear to play an essential role because lipoedema symptoms often debut during hormone alteration periods, such as during puberty, pregnancy, or menopause [7]. It is also assumed that lipoedema has a genetic predisposition as patients often have a lipoedema-affected female relative [8]. Moreover, lipoedema fat differs from typical fat in its structure and metabolism [9], with the loose connective tissue involved in lipoedema being characterized by hypertrophic adipocytes, fibrosis, and inflammatory angiogenesis [10]. Currently, no specific biomarkers for diagnosing lipoedema are available, with it being confirmed solely through the use of a thorough medical history-taking and a comprehensive physical examination that includes both inspection and palpation [6].

Lipoedema fat is most common in the lower limbs but can occur in any subcutaneous adipose tissue throughout the body. Depending on fat location, the disease is classified into five different types. Type 1 refers to pelvis, buttocks, and hips; type 2 includes from the buttocks to knees; type 3 is from buttocks to ankles; type 4 is the arms; and type 5 includes the lower legs [1]. In addition to these types, there is also a classification of its three stages according to the disease progression, which is determined through a clinical examination. Stage 1 refers to a normal skin surface but enlarged hypodermal subcutaneous adipose tissue, stage 2 includes indentations of the skin and underlying larger mounds of subcutaneous adipose tissue, and stage 3 involves large extrusions of tissue that cause gross deformations. Concomitant with progression in these three stages, women with lipoedema are also at risk of developing lipo-lymphoedema (i.e., clinically identifiable lymphoedema) [11].

The leading symptom of lipoedema is pain. Other common symptoms include numbness, easy bruising, fatigue, muscle weakness, and feeling generally swollen. As the disease progresses, leg heaviness may increase and cause impaired mobility [12, 13]. Currently, there is no cure for lipoedema. Present treatment focus on symptom relief, with management options including patient education, psychosocial support, promoting self-care, and the encouraging of a healthy lifestyle. More conservative treatment includes manual lymphatic drainage and compression therapy to lessen any pain and swelling [6]. Liposuction is one surgical option that has been shown to reduce bruising, immobility, and pain in a way that then improves quality of life. However, further randomized studies assessing the clinical benefits and long-term outcomes of liposuction are needed [14]. Having lipoedema has also been described as burdensome due to the resulting deficits in the ability to function physically and socially, the lengthy-time for receiving a diagnosis, experiences of being fat-shamed, and receiving unsupportive and misguided advice from healthcare professionals [15]. In addition, diverse and multiple comorbidities, such as obesity, hypothyroidism, migraines, and depression, are more common among lipoedema patients than in non-lipoedema populations [16].

Health-related quality of life (HRQOL) refers to how health affects an individual’s perceived well-being and the ability to function in the physical, mental, and social domains of life [17]. The few studies exploring lipoedema’s effect on HRQOL have shown that this disease has a significantly negative impact on health and social functioning [18]. A study focusing on the psychological status and quality of life of people with lipoedema found that those with higher symptom severity reported lower levels of HRQOL [19]. Additionally, a lower level of HRQOL was predicted by lower mobility, depression, and higher appearance-related distress [20, 21].

Women with lipoedema often describe experiences of unhelpful and misguided advice from healthcare professionals, resulting in them struggling to, by themselves, gather information in order to understand and manage their health problems [15]. The concept of sense of coherence (SOC), originated from Aron Antonovsky, reflects a person’s capacity to manage tension and identify and mobilize their internal and external resources in a health-promoting manner. SOC is crucial in maintaining good health and in coping with stressful life events, such as chronic disorders [22]. Moreover, a strong SOC is associated with better-perceived health and a higher quality of life [23, 24]. However, to our knowledge, no previous study has examined SOC among women with lipoedema.

While biomedical research on lipoedema has increased over the last decade, there is still a knowledge gap in how affected women experience their health and functioning and what inner health resources they possess. Therefore this national survey examined health, HRQOL, and SOC among women with lipoedema.

## Methods

### Study design, setting, and participants

A national cross-sectional study using an online survey was carried out. Participants were recruited from all Lipoedema Association groups in Sweden.

Before conducting the full-scale data collection, the survey was tested by fifteen women with lipoedema that were recruited from four Lipoedema Association groups. The aim here was to ensure that the questions were understandable and were presented consistently, to explore variability in responses, and to estimate how much time was required to respond to the survey. Ten participants provided written feedback to the authors in a structured questionnaire, resulting in minor corrections and clarifications to the final survey.

The data collection in the final survey was conducted between June and September 2021. Based on 80% power, a *p*-value set at < 0.05, and a medium effect size d = 0.308, a sample size of 166 participants in each group was strived for [25]. The corresponding author coordinated the survey distribution in cooperation with the board members from all Lipoedema Associations groups. An email (followed by two reminders) that included study information, an invitation to participate, an informed consent form, and a link to the survey was sent out to approximately 700 members of all Lipoedema Association groups in Sweden. Member categories were women with lipoedema, support members (such as family members), and healthcare professionals with a special interest in lipoedema. However, our inclusion criteria were being female, aged 18 years or older, have a confirmed lipoedema diagnosis, or have lipoedema symptoms. In total, 245 women with lipoedema completed the survey.

### Measurements

#### Sociodemographic and health data

The survey began with questions on sociodemographic factors, followed by those on health-related matters, including self-reported lipoedema characteristics (i.e. lipoedema type, stage, onset, and diagnosis), symptom severity, and comorbidities. The authors developed these questions based on published scholarly lipoedema literature, as well as in collaboration with a physician working in primary public healthcare with clinical experience of lipoedema and two members from a Lipoedema Association group (one woman with lipoedema and one spouse). Questions on lipoedema type and stage were presented with illustrated images and explanatory text. Participants reported any comorbidities by filling in pre-specified health problems and diseases and, as a complement, participants could also respond using free text. Symptom severity was measured by a 47-item questionnaire wherein participants were asked to rate their experiences of various listed lipoedema-associated symptoms over the previous month on a 4-point Likert scale. The questionnaire also included pain in bodily areas where lipoedema does not typically occur (i.e., hands, neck, shoulders, and back) to gain a more comprehensive picture of pain. The overall question was *‘During the past month, to what extent have you experienced this symptom?’* with the following answer options being presented: 1= *‘I have not experienced this symptom at all’*, 2= *‘I have been slightly affected by this symptom’*, 3= *‘I have been moderately affected by this symptom’,* and 4= *‘I have been severely affected by this symptom’.*

#### RAND-36

Respondents’ HRQOL was measured using the Swedish version of the RAND-36 [26], which comprises 36 items on health. Herein, 35 of these items measure eight conceptual health attributes (i.e. subscales), including physical functioning, physical role functioning, pain, general health, energy/fatigue, social functioning, emotional role functioning, and emotional well-being. In addition, there is a single item used for measuring perceived health changes in the past year. Each scale is presented with a score from 0 to 100, wherein a higher score indicates an overall better HRQOL [17]. The RAND-36 is recommended as a generic measurement tool for lipoedema [27]. Permission for its use and manuals for the Swedish version were obtained from a regional Swedish register center [28]. In addition, RAND-36 reference data for an age-matched general Swedish female population was obtained from the corresponding author in the published Mid-Swed Health Survey [29].

#### SOC-13

SOC was measured using the Swedish version of the SOC-13. The SOC-13 is universal and has been validated for use among different cultures, ages, and diseases, with it comprising 13 items across three sub-scales, including comprehensibility, manageability, and meaningfulness. Each item consists of a Likert scale ranging from 1 to 7, which results in a total score range of 13–91. A higher score represents a higher overall SOC [30]. Permission to use, including a codification file for the Swedish version of the SOC-13, was obtained from the Society for Theory and Research on Salutogenesis [31].

### Statistical analyses

Statistical analyses were conducted using IBM SPSS Statistics version 27.0 and Stata version SE 15.1.2017.

Data on respondents’ sociodemographic and lipoedema characteristics were compiled along with descriptive statistics. An exploratory factor analysis was then performed to examine the underlying correlation patterns for the items in the 47-item symptom severity questionnaire. A principal component analysis was then conducted using suppressed coefficients below 0.5 and a varimax rotation. Items with communalities lower than 0.5, as well as those loading at more than one component or no component, were manually extracted, which resulted in 41 remaining items. The sampling adequacy for the factorability was measured using the Kaiser-Meyer-Olkin (KMO) showing 0.908 and a Bartlett test of sphericity showing *p* = 0.00. A nine-factor solution was then extracted (Additional file 1), with total variance explained at 73.9%, which, when combined with a visual inspection of the rotated component matrix, was considered a good fit for the data. Data analyses were performed by calculating the items’ scores across each factor.

The RAND-36’s generated data on the ordinal level for each item, which in the next step were recoded, were calculated and averaged into subscale scores by using a standard scoring algorithm. It is recommended that these scores are treated as a new ordinal scale [25]. Further, scale scores were only calculated if participants had responded to at least half of the items in each scale. If this was true, any single missing values were imputed using the item mode value based on the whole group. SOC scores were then calculated by adding the points marked for each item (items with reversed scores were also converted). Questionnaires with more than three items unanswered were excluded. Cronbach’s alpha was used to calculate item-scale correlations, showing 0.80–0.92 in the RAND-36 subscales and 0.71–0.77 in the SOC subscales.

Differences between groups were analysed using the non-parametric statistics tests Mann Whitney and Kruskal Wallis. A chi-squared test was used when testing for significant differences between lipoedema stages and symptom severity. When comparing RAND-36 mean scores between this study sample and the aggregated data from a general Swedish female population the parametric independent t-test was used. The significance level set for all these tests was *p* < 0.05.

## Results

### Sociodemographic and lipoedema characteristics

Participants’ sociodemographic and self-reported lipoedema characteristics are listed in Table 1. The majority (62.9%) of the women were age 40–59 years. Over half of the sample, 54.3%, reported being in lipoedema stage 3. The most common lipoedema type (not presented in Table 1) was a combination of type 3 (buttocks to ankles) and type 4 (arms), which was reported among 58.7% of participants. Further, 33 of the women reported having lipoedema in other body locations than those stated in the current classification types, with approximately 10% of all participants responding (by free-text answers) as having lipoedema in the abdomen. Additionally, 76.2% of the women in this sample had been diagnosed with lipoedema by a healthcare professional. Despite that 169 women (69%) reported that their lipoedema onset had occurred before the age of 30, only three of them (1.6%) was diagnosed by a healthcare professional prior to this age. The most common age for being diagnosed with lipoedema was 50–59 years (34.9%), which indicates an extensive diagnostic delay.

**Table 1:** Descriptive statistics of participants’ sociodemographic and self-reported lipoedema characteristics

Variables	Frequency (n)	Percentage (%)
Age (years)	245	
18–39	16	6.5
40–49	57	23.3
50–59	97	39.6
60–69	45	18.4
70 and older	30	12.2
Educational level	245	
Mandatory/High school	64	26.1
Upper secondary/University	181	73.9
Occupation^a^	244	
Working full-time	132	54.1
Working part-time	38	15.6
Studying	9	3.7
On sick leave (wholly or partially)	33	13.5
Unemployed	9	3.7
Retired	48	19.7
Age at lipoedema onset (years)	245	
11 or younger	19	7.8
12–17	99	40.4
18–29	51	20.8
30–39	26	10.6
40–49	24	9.8
50 or older	26	10.6
Confirmed diagnosis	244	
Yes	186	76.2
No/Do not know	58	23.8
Age at diagnosis (years)	186	
12–29	3	1.6
30–39	30	16.1
40–49	52	28.0
50–59	65	34.9
60 or older	36	19.4
Lipoedema stage^b^	245	
Stage 1	23	9.4
Stage 2	81	33.1
Stage 3^c^	133	54.3
Do not know	8	3.3

^a^30 participants responded to more than one response option here. Among those, the most common combination (*n* = 7) included those working part-time and on sick leave part-time

^b^Participants who responded to being in more than one stage were then grouped into their most advanced stage

^c^Stage 3 also includes participants who reported being in stage 4 (*n* = 11), which was classified as lipoedema stage 3 with secondary lymphedema

### Comorbidities

**Fig. 1:**
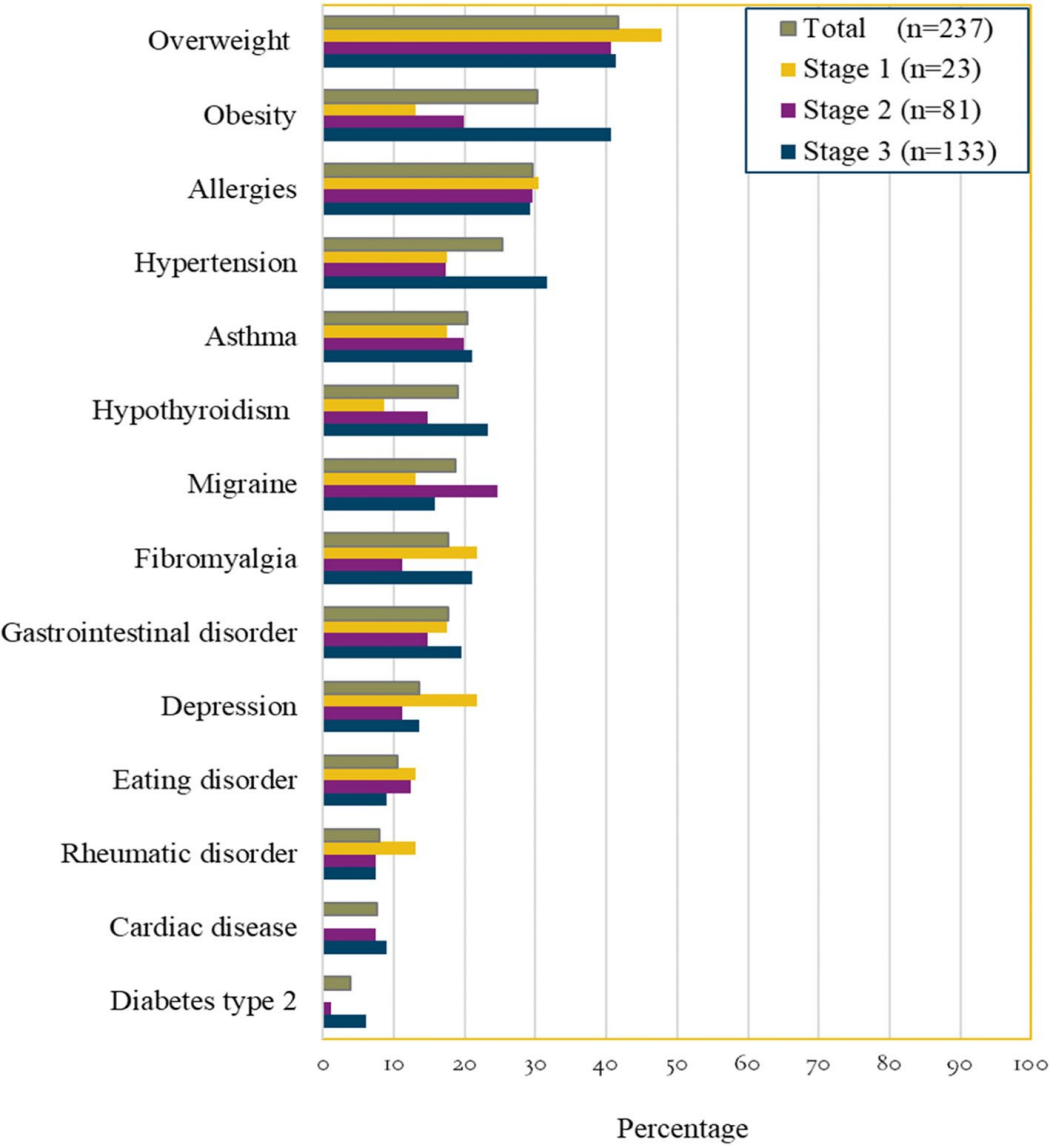
Illustrates the prevalence of comorbidities in total and in relation to each lipoedema stage

All participants, except 16 women (6.5%), reported having at least one comorbidity. Figure 1 presents the comorbidities in total and in each of the lipoedema stages. In our sample, the most common comorbidities were overweight and obesity, reported among 41.7 and 30.2% of the participants, respectively. Nearly one-fifth of all women had hypothyroidism, which was more common in more progressive stages, with it affecting 23.3% of women in lipoedema stage 3 compared to 8.7% of those in stage 1. Furthermore, 25.3% reported having hypertension, with the highest prevalence (31.6%) of this being observed among women in lipoedema stage 3. Over 17% of the women had fibromyalgia, and 13.5% reported having depression. About 20% (*n* = 45) of all women had other comorbidities than those listed in Fig. 1; among these, the most mentioned in their free-text answers were arthrosis (*n* = 8) and Ehler Danlos syndrome (*n* = 4).

### Symptom severity in lipoedema stages

**Fig. 2:**
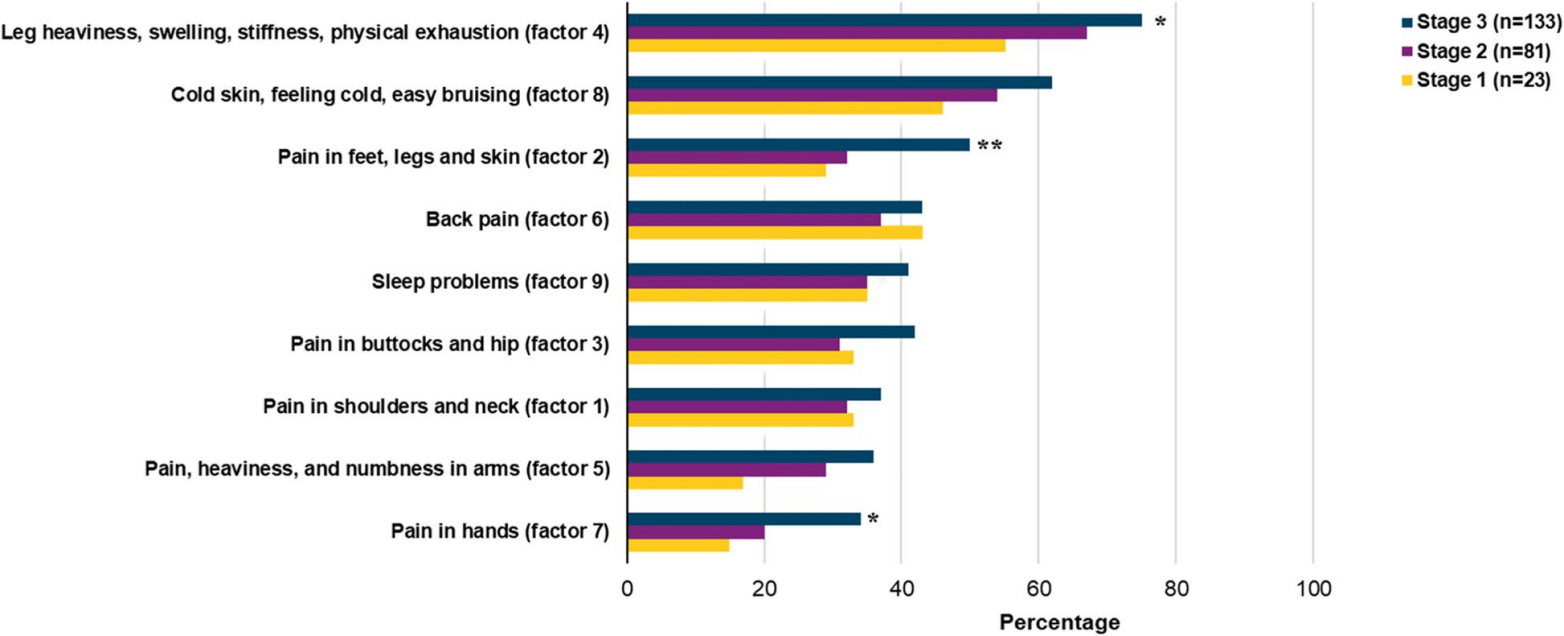
The percentage of symptoms (in factors) scored as ‘moderate or severe’ in each lipoedema stage. * *p* value < 0.05. Significant difference between stages 1 and 3. ** p value < 0.01. Significant difference between stages 2 and 3

Figure 2 illustrates the percentage of participants responses to ‘*I have been moderately/ I have been severely affected by this symptom*’ in each factor (Additional file 1) in relation to respondents’ lipoedema stage. This figure shows that moderate and severe lipoedema symptoms were reported across all stages, but most often in stage 3. Moderate or severe symptoms in terms of leg heaviness, swelling, stiffness, and feeling exhausted at light physical exertion (factor 4) were significantly more common among women in lipoedema stage 3 than those in stage 1 (*p = <* 0.05). Easily bruising was reported among 97.5% of participants.^1^ Moreover, among women in stage 3, we found that 62% of the questions on cold skin, feeling cold, and easily bruising (factor 8) were responded to as being moderate or severe, compared to 46% in stage 1. Almost all participants (98.7%) reported having pain^1^. Pain in feet, leg, and skin (factor 2) were more frequently reported as being moderate or severe in stage 3 than in stage 2, with responses coming to 50 and 32%, respectively (*p* = < 0.01). Pain in the back (factor 6), shoulders and neck (factor 1), buttocks and hips (factor 3), and sleep problems (factor 9) were in general equally reported on as either moderate or severe in all stages. Pain in hands (factor 7) was significantly more often reported as being moderate or severe among women in stage 3 than those in stage 1 (*p = <* 0.05). Moderate or severe symptoms of hypermobile joints^2^ were reported in 39% of stage 1 respondents, 49% of those in stage 2, and 58% of those in stage 3.

### Health-related quality of life

Table 2 shows the HRQOL scores across the various subscales among participants. For the whole sample, the lowest scores were found in physical role-functioning (mean 38.2/SD 40.5), which reflects difficulties with work or other daily activities due to physical health. Conversely, the highest scored health scale was emotional well-being, with a mean of 64.9 (SD 19.7).

**Table 2:** Health-related quality of life (RAND-36) among women with lipoedema grouped into sociodemographic and lipoedema characteristics

Variable	Physical functioning mean (SD)	Role functioning/ physical mean (SD)	Pain mean (SD)	General health mean (SD)	Energy/ fatigue mean (SD)	Social functioning mean (SD)	Role functioning/ emotional mean (SD)	Emotional well-being mean (SD)	Health change mean (SD)
Whole sample (*n* = 245)	55.1 (27.6)	38.2 (40.5)^a^	43.9 (24.0)	43.7 (23,1)^a^	40.5 (22.6)^a^	56.5 (27.6)	58.3 (42.4)^b^	64.9 (19.7)^a^	41.7 (24.5)^a^
Age in years (*n* = 245)									
18–39 (*n* = 16)	75.3 (12.9)	56.3 (40.3)	46.7 (21.8)	44.4 (21.1)	38.4 (16.0)	63.3 (21.6)	81.3 (32.1)	63.0 (16.3)	50.0 (18.3)
40–49 (*n* = 57)	66.0 (25.8)	45.6 (44.1)	52.5 (26.4)	45.3 (27.4)	44.4 (22.3)	59.4 (29.4)	53.8 (44.0)	65.5 (22.2)	47.4 (27.8)
50–59 (*n* = 97)	54.1 (28.2)	38.7 (41.0)	40.6 (24.2)	42.3 (21.3)	37.2 (23.8)	54.0 (28.7)	59.4 (41.1)^a^	64.2 (19.7)	38.5 (24.6)^a^
60–69 (*n* = 45)	47.1 (23.0)	32.2 (35.6)	38.2 (18.0)	42.3 (20.8)	38.0 (22.4)	58.3 (25.0)	64.4 (41.7)	65.3 (16.9)	38.9 (19.6)
70+ (*n* = 30)	38.7 (27.7)	21.6 (32.5)^a^	44.9 (24.3)	47.1 (25.2)^a^	48.8 (20.0)^a^	52.9 (27.4)	41.4 (44.3)^a^	66.8 (21.5)^a^	40.8 (25.8)
*p* value	**<.001**	**.032**	**.046**	.933	.140	.491	**.031**	.861	.062
Educational level (*n* = 245)									
Mandatory/High school (*n* = 64)	46.3 (26.9)	30.5 (40.5)	39.5 (21.5)	40.9 (22.2)	37.2 (22.2)	49.0 (28.1)	49.2 (43.5)^a^	58.4 (21.2)	39.1 (22.7)
Upper secondary/University (*n* = 181)	58.2 (27.3)	41.0 (40.2)^a^	45.4 (24.7)	44.7 (23.4)^a^	41.6 (22.6)^a^	59.2 (27.1)	61.5 (41.7)^a^	67.2 (18.7)^a^	42.6 (25.2)^a^
*p* value	**.003**	.051	.112	.309	.211	**.012**	**.047**	**.002**	.329
Lipoedema onset (*n* = 243)									
Age 17 years or younger (*n* = 118)	58.8 (26.9)	41.1 (42.2)	46.3 (23.8)	45.8 (23.5)	43.6 (22.4)	62.0 (27.6)	66.9 (40.5)	68.3 (19.0)	47.0 (23.7)^a^
Age 18 years or older (*n* = 125)	52.2 (27.8)	36.1 (38.8)^a^	42.0 (24.0)	42.0 (22.8)^a^	37.7 (22.5)^a^	52.1 (26.5)	50.9 (42.5)^b^	62.3 (19.5)^a^	37.2 (24.3)
*p* value	.064	.577	.248	.239	.078	**.004**	**.004**	**.017**	**.002**
Lipoedema onset during hormonal changes (*n* = 244)									
Puberty (*n* = 108)	59.6 (28.8)	40.5 (43.2)	45.8 (25.8)	46.4 (23.9)	43.2 (23.4)	60.4 (28.0)	63.9 (41.8)	68.5 (19.6)	47.2 (24.6)^a^
Pregnancy (*n* = 46)	53.9 (26.3)	37.0 (39.0)	41.7 (20.4)	44.1 (23.7)	34.8 (22.1)	50.5 (27.2)	57.2 (41.4)	60.1 (19.4)	38.0 (28.7)
Menopause (*n* = 38)	44.7 (21.7)	27.7 (32.7)^a^	36.1 (19.4)	38.0 (19.6)^a^	35.0 (22.2)^a^	50.3 (22.4)	48.6 (39.0)^a^	62.2 (17.3)^a^	34.9 (19.7)
Other timepoint (*n* = 52)	54.1 (28.8)	41.3 (40.8)	47.6 (25.3)	42.1 (23.3)	43.8 (20.5)	58.4 (29.9)	53.6 (46.2)^a^	63.9 (21.1)	38.5 (21.8)
*p* value	**.023**	.436	.140	.328	.100	.066	.219	.060	**.026**
Confirmed diagnosis (*n* = 244)									
Yes (*n* = 186)	54.9 (28.2)	37.5 (41.3)	42.8 (24.7)	43.4 (24.0)	41.3 (23.2)	56.3 (28.7)	58.9 (42.6)^b^	64.9 (19.6)	43.2 (25.3)^a^
No/Do not know (*n* = 58)	56.3 (25.8)	41.2 (37.9)^a^	47.4 (21.5)	44.2 (20.1)^a^	37.5 (20.2)^a^	57.1 (24.1)	55.7 (42.1)	64.8 (20.3)^a^	36.2 (21.0)
*p* value	.786	.380	.113	,740	.235	.997	.680	.974	.075
Age at diagnosis in years (*n* = 186)									
12–39 (*n* = 33)	62.3 (24.1)	43.9 (43.8)	47.9 (26.7)	41.1 (24.4)	43.2 (20.3)	64.4 (25.8)	73.7 (38.0)	66.9 (20.7)	48.5 (26.5)
40–49 (*n* = 52)	63.6 (30.8)	50.4 (45.5)	48.3 (27.3)	47.3 (26.4)	43.9 (23.4)	56.0 (31.1)	56.9 (43.9)^a^	64.7 (21.7)	44.7 (24.9)
50–59 (*n* = 65)	52.4 (26.1)	29.6 (36.7)	37.7 (23.4)	40.5 (22.3)	35.0 (24.8)	52.1 (28.2)	56.4 (42.0)	63.6 (16.7)	41.4 (26.8)^a^
60 + (*n* = 36)	40.0 (25.5)	27.1 (35.5)	39.2 (19.2)	45.3 (23.3)	47.2 (20.5)	56.6 (28.1)	52.4 (44.5)^a^	65.9 (21.0)	39.6 (21.9)
*p* value	**<.001**	**.041**	.076	.377	.076	.190	.162	.525	.260
Lipoedema stage (*n* = 237)									
Stage 1 (*n* = 23)	60.9 (30.2)	48.9 (46.1)	49.6 (25.2)	48.7 (23.7)	41.1 (27.1)	64.1 (26.7)	65.2 (40.8)	64.3 (21.5)	43.5 (21.6)
Stage 2 (*n* = 81)	68.9 (22.8)	49.1 (40.2)	50.0 (21.2)	48.3 (23.5)	42.6 (22.5)	60.0 (26.2)	55.4 (40.4)^a^	63.8 (17.4)	46.9 (22.8)
Stage 3 (*n* = 133)	45.9 (26.2)	30.7 (38.4)^a^	38.6 (24.6)	39.8 (22.7)^a^	39.4 (22.4)^a^	53.2 (28.4)	58.8 (43.9)^a^	65.3 (20.8)^a^	38.3 (25.9)^a^
*p* value	**<.001**	**.003**	**<.001**	**.016**	.490	.082	.521	.558	**.030**

Bold: *p* value < 0.05. ^a^ = 1 missing, ^b^ = 2 missing

Women in lipoedema stage 3 reported significantly lower scores in physical health compared to those in stages 1 and 2, including in terms of physical functioning; for example, dressing or walking on stairs (*p* < .001), physical role-functioning including work-related activities (*p =* .003), more severe and limiting pain (*p* < .001), and worse general health (*p =* .016), as well as in health changes (i.e. a deterioration in health over the past year (*p =* .030). Worse physical health was also found among women at age 70 or older in the scales of physical functioning (*p* < .001) and physical role-functioning (*p =* .032) compared to those in all younger age groups. In addition, women who had experienced lipoedema onset as adults (i.e. age 18 or older) reported poorer mental health than those with lipoedema onset in adolescence (age 17 or younger), with significantly lower scores in social functioning (*p =* .004), emotional role-functioning (*p =* .004), emotional wellbeing (*p =* .017), and overall lower scores in health changes over the past year (*p =* .002). Moreover, women with a mandatory/high school education scored lower in social functioning (*p =* .012), emotional role-functioning (*p* = .047), and emotional wellbeing (*p =* .002) compared to those with upper secondary/university education. Finally, no significant differences in HRQOL were found between women with a confirmed or unconfirmed lipoedema diagnosis.

Compared to an age-matched general Swedish female population, women with lipoedema scored significantly lower in all HRQOL scales— except for emotional role-functioning among the age groups 30–39, wherein our study sample reported higher scores (Table 3). Overall, in most age-groups, women with lipoedema scored approximately 25–35 points lower in both their physical and mental health. The largest difference in points was found in physical role functioning, wherein women with lipoedema in age groups 60–69 and 70–79 scored approximately 43 points lower than the same age groups in the general population (*p* < .001). The lowest differences in points were observed in emotional wellbeing, wherein women with lipoedema scored approximately 10 points lower than the general female population.

**Table 3:** RAND-36 scores for women with lipoedema (L) compared to a general Swedish female population (S)

Age (years)	Sample (n)	Physical functioning mean (SD) *p* value	Role functioning/ physical mean (SD) *p* value	Pain mean (SD) *p* value	General health mean (SD) *p* value	Energy/ fatigue mean (SD) *p* value	Social functioning mean (SD) *p* value	Role functioning/ emotional mean (SD) *p* value	Emotional wellbeing mean (SD) *p* value
30–39	L (15)	75.3 (13.4)	55.0 (41.4)	46.0 (22.3)	42.7 (20.7)	36.7 ^1^(14.9)	62.5 (22.1)	82.2 (33.0)	62.7 (16.8)
	S (291)	90.6 (18.2)^a^	79.9 (34.7)^b^	78.9 (23.6)	70.3 (21.4)	53.8 (21.1)^a^	81.0 (22.9)	70.5 (38.9)^c^	72.5 (18.1)^a^
*p* value		**<.001**	**.037**	**<.001**	**<.001**	**<.001**	**.006**	.203	**.044**
40–49	L (57)	66.0 (25.8)	45.6 (44.1)	52.5 (26.4)	45.3 (27.4)	44.4 (22.3)	59.4 (29.4)	53.8 (44.0)	65.5 (22.2)
	S (232)	90.3 (14.3)	77.7 (31.3)	77.1 (20.7)	70.5 (17.8)^a^	57.3 (17.1)	82.0 (19.0)	77.2 (30.9)^a^	74.5 (13.7)
*p* value		**<.001**	**<.001**	**<.001**	**<.001**	**<.001**	**<.001**	**<.001**	**.005**
50–59	L (97)	54.1 (28.2)	38.7 (41.0)	40.6 (24.2)	42.3 (21.3)	37.2 (23.8)	54.0 (28.7)	59.4 (41.1)^a^	64.2 (19.7)
	S (233)	80.9 (21.9)	73.3 (35.2)^c^	69.9 (35.2)^a^	65.6 (21.8)^a^	58.2 (22.4)^a^	79.7 (24.0)^a^	75.1 (33.9)^c^	75.2 (17.7)^a^
*p* value		**<.001**	**<.001**	**<.001**	**<.001**	**<.001**	**<.001**	**.001**	**<.001**
60–69	L (45)	47.1 (23.0)	32.2 (35.6)	38.2 (18.0)	42.3 (20.8)	38.0 (22.4)	58.3 (25.0)	64.4 (41.7)	65.3 (16.9)
	S (305)	79.7 (23.6)^a^	75.2 (39.6)^b^	70.7 (27.2)^b^	67.7 (22.8)^c^	65.5 (23.8)^b^	84.7 (23.8)	81.1 (35.9)^c^	78.3 (20.9)^b^
*p* value		**<.001**	**<.001**	**<.001**	**<.001**	**<.001**	**<.001**	**.014**	**<.001**
70–79	L (25)	39.6 (28.0)	22.9 (32.9)^a^	43.7 (24.8)	45.4 (26.9)^a^	47.9 (21.1)^a^	52.5 (29.3)	38.9 (46.8)^a^	64.3 (21.6)^a^
	S (313)	69.9 (32.7)^a^	65.7 (50.5)^f^	67.6 (33.6)^a^	64.1 (26.3)^d^	65.4 (27.3)^a^	82.7 (29.1)	76.3 (45.3)^e^	76.7 (23.7)^c^
*p* value		**<.001**	**<.001**	**<.001**	**.003**	**<.001**	**<.001**	**<.001**	**.010**

Bold: *p* value ≤0.05. a = 1 missing, b = 2 missing, c = 3 missing, d = 4 missing, e = 9 missing, f = 11 missing

Scores in age groups 18–29 and 80+ years is not presented due to a low number of lipoedema participants (≤5) in these groups

### Sense of coherence

A stronger SOC was primarily found to be associated with sociodemographic variables and less so with lipoedema characteristics. Women aged 70 years or older had significantly higher scores in total SOC (*p =* .010), comprehensibility (*p* = .015), and manageability (*p =* .003) compared to those in younger age groups. Furthermore, participants with a higher educational level (secondary school/university) scored higher for total SOC (*p =* .010), meaningfulness (*p* = .003), and manageability (*p =* .032) compared to those who had only completed mandatory/high school education. No statistically significant differences in total SOC or the associated subscales were found to be related to lipoedema stage, lipoedema onset related to hormonal changes, or age at lipoedema onset, except for lower scores in meaningfulness among participants with lipoedema onset as adults compared to those whose onset was in adolescence (*p* = .017). However, the highest total SOC (mean = 66.2) was observed among women diagnosed with lipoedema at age 60 or older. Additionally, this group scored higher in manageability and comprehensibility than did the younger age groups (Table 4).

**Table 4:** Sense of coherence (SOC-13) grouped into sociodemographic and lipoedema characteristics

Variable	SOC total mean (SD)	Comprehensibility mean (SD)	Meaningfulness mean (SD)	Manageability mean (SD)
Women with lipoedema (*n* = 241)	60.3 (14.3)	22.8 (6.4)	19.8 (4.8)	17.6 (5.0)
Age in years (*n* = 241)				
18–39 (*n* = 16)	60.5 (11.3)	23.0 (5.6)	20.1 (4.7)	17.4 (3.7)
40–49 (*n* = 57)	58.6 (14.4)	21.8 (6.0)	20.1 (5.0)	16.6 (5.0)
50–59 (*n* = 95)	57.6 (14.2)	21.8 (6.5)	19.1 (4.9)	16.8 (4.8)
60–69 (*n* = 44)	64.6 (13.7)	24.8 (6.1)	20.4 (4.5)	19.4 (4.8)
70+ (*n* = 29)	65.6 (14.8)	25.0 (6.6)	20.9 (4.5)	19.7 (5.3)
*p* value	**.010**	**.015**	.304	**.003**
Educational level (*n* = 241)				
Mandatory or High school (*n* = 64)	56.5 (15.6)	21.6 (6.8)	18.3 (5.1)	16.6 (5.2)
Secondary school or University (*n* = 177)	61.6 (13.6)	23.2 (6.1)	20.4 (4.5)	18.0 (4.8)
*p* value	**.010**	.067	**.003**	**.032**
Lipoedema onset (*n* = 239)				
Age 17 years or younger (*n* = 116)	62.1 (13.0)	23.6 (5.8)	20.7 (4.6)	17.8 (4.7)
Age 18 years or older (*n* = 123)	58.7 (15.2)	22.1 (6.7)	19.1 (4.8)	17.5 (5.1)
*p* value	.115	.066	**.017**	.823
Lipoedema onset during hormonal changes (*n* = 240)				
Puberty (*n* = 107)	61.7 (13.3)	23.4 (5.9)	20.5 (4.7)	17.7 (4.7)
Pregnancy (*n* = 45)	57.5 (13.5)	21.6 (6.2)	18.5 (4.7)	17.3 (4.7)
Menopause (*n* = 37)	62.1 (15.1)	23.7 (6.6)	19.9 (4.4)	18.5 (5.2)
Other timepoint (*n* = 51)	58.4 (16.4)	21.8 (7.1)	19.5 (5.2)	17.1 (5.5)
*p* value	.389	.268	.141	.681
Confirmed diagnosis (*n* = 240)				
Yes (*n* = 183)	60.0 (14.6)	22.8 (6.5)	19.8 (4.8)	17.5 (5.1)
No/Do not know (*n* = 57)	61.1 (13.5)	23.0 (5.8)	20.0 (4.8)	18.1 (4.6)
*p* value	.766	.983	.731	.499
Age at diagnosis (*n* = 183)				
12–39 years old (*n* = 33)	60.3 (13.8)	23.2 (5.9)	20.3 (5.5)	16.7 (4.8)
40–49 years old (*n* = 51)	58.4 (14.6)	22.0 (6.5)	19.8 (4.8)	16.6 (5.0)
50–59 years old (*n* = 64)	57.7 (14.5)	21.6 (6.4)	18.9 (4.8)	17.2 (5.0)
60 + years old (*n* = 35)	66.2 (14.6)	25.5 (6.9)	20.7 (4.0)	20.0 (5.1)
*p* value	**.021**	**.015**	.230	**.009**
Lipoedema stage (*n* = 233)				
Stage 1 (*n* = 23)	59.9 (16.2)	22.1 (6.6)	20.3 (4.9)	17.4 (5.7)
Stage 2 (*n* = 81)	60.1 (14.2)	22.5 (6.4)	19.7 (4.6)	17.9 (5.0)
Stage 3 (*n* = 129)	60.2 (14.1)	23.0 (6.4)	19.8 (4.9)	17.4 (4.9)
*p* value	.937	.732	.850	.786

Bold: *p* value **<** 0.05

## Discussion

This is the first national study on health, HRQOL, and SOC in a Swedish female lipoedema population. The main findings were that 1) lipoedema, even in early stages, is associated with several health problems and a lower overall HRQOL and 2) that women with lipoedema most often wait decades before being correctly diagnosed by the healthcare system. These findings are important to discuss both separately and in relation to one another.

This study found that comorbidities among the participants were common. The fact that lipoedema is associated with diverse and multiple comorbidities has also been presented in previous studies [16, 32]. Most common comorbidities observed in this study were being overweight and having obesity, which then sheds a new light onto the challenges often faced by women with lipoedema regarding weight loss. First, lipoedema fat is resistant to weight loss, meaning that diets and exercise have minimal effects [33]. Second, our study shows that the highest prevalence of obesity (approximately 40%) was found among women with lipoedema stage 3, which is the stage wherein participants reported the lowest physical functioning and worst levels of pain. Based on these findings, we can assume that losing weight in any non-lipoedema fat may be a challenge due to disabilities in mobility and physical functioning. Third, repeated attempts to lose weight can cause a shift in metabolism with a decrease in energy expenditure, which may then lead to weight regain or a spiral of weight gain [34]. Nevertheless, due to its overall positive effect on health, healthcare professionals should encourage and support patients with lipoedema in striving for a lifestyle that includes healthy eating and physical activity [35].

Additionally, approximately 20% of participants reported having hypothyroidism, a higher prevalence than the estimated prevalence of 7% in the general Swedish female population [36]. A similar prevalence of hypothyroidism among lipoedema patients has been described previously [37]. Although there is an association between hypothyroidism and obesity [38], there is still a lack of evidence to explain the high prevalence of the former among women with lipoedema [32]. Because hypothyroidism, like lipoedema, may cause health issues such as fatigue, weight gain, feeling cold, and sleep problems [39] we recommend health care professionals to bring attention to, and to evaluate thyroid function among women with lipoedema. Furthermore, over 17% of participants in this study reported having fibromyalgia, which is a higher prevalence than reported in females in the general populations that ranges from 2.4 to 6.8% [40]. A recent work claims that more attention should be paid to the fact that fibromyalgia and lipoedema (especially lipoedema stage 1) share several clinical characteristics and may be challenging to distinguish [41].

Notably, this study found that women with lipoedema onset in adolescence scored better in the mental health HRQOL scales than those with lipoedema onset in adulthood. This result was surprising because there is a clear association between dissatisfaction in pubertal body changes and decreased body esteem among teenagers, and lower psychological functioning in adulthood, especially among girls and women with a higher body mass index [42]. Because our data cannot draw conclusions on this or explain this result we can only, based on previous research, assume that psychosocial factors, such as social support and the consequences of stigma on general health and social functioning [43, 44] also may play an essential role when living with lipoedema, which should be further studied.

Furthermore, this study found that women with lipoedema reported a significantly lower HRQOL than does the general female population. Among participants in this study, the lowest points in HRQOL were observed in physical role functioning, indicating that women with lipoedema are heavily limited in their daily work or activities due to poor physical health. These results emphasize the findings from a previous study wherein women with lipoedema described their bodies as painful, burdensome, unreliable, and unpredictable [15]. Moreover, the burden of comorbidities in patients with lipoedema needs to be considered as a critical compounding factor to HRQOL [16]. Therefore, healthcare professionals must be aware of and manage the complexity of lipoedema and any comorbidities and their negative impact on patients’ health status and quality of life.

Although moderate and severe lipoedema symptoms and poor physical health were reported in all stages, women in lipoedema stage 3 primarily reported on complaints of heaviness, swelling, feeling cold, easy bruising, and hypermobility in joints. These symptoms tend to increase as lipoedema progress into higher stages and are assumed to be associated with changes in loose connective tissue such as dysfunctional blood vessels, a loss of elasticity in lipoedema tissue, and an excess of interstitial fluid [45]. In this study, we paid particular attention to pain that was reported as moderate or severe, and therefore more likely to affect health and daily life. We found that moderate and severe pain were reported across all lipoedema stages. Our symptom severity questionnaire, included questions on pain location and pain severity, while information on pain nature were not elucidated upon. However, one previous work does describe lipoedema pain as heterogeneous (i.e. it can be dull, heavy, burning, and pressing) [13]. Herein, how pain affects HRQOL depends on its extent, intensity, duration, underlying diseases, and inner health resources [46]. Moreover, chronic pain and disability can be explained as a complex interaction among biological (sex, genetics, hormones), psychological (coping skills, personality), and social (social expectation, social support) factors [47]. Therefore, when supporting women with lipoedema in managing their pain and improving their HRQOL, a careful assessment of the exact nature of their pain and the application of a holistic approach in healthcare is necessary. Furthermore, a crucial area in future research would be the evaluating of various treatment plans for lipoedema pain.

Even though no statistically significant difference was found in the HRQOL between groups of those who had actually received a lipoedema diagnosis versus those who had not, non-diagnosed women scored 7 points lower in health changes over the past year (i.e. a decrease in health) than did those with a confirmed lipoedema diagnosis. Because the minimal clinical importance for the RAND-36 has been proposed as a range of 3 to 5 points [48], this result could still be considered important, indicating that lipoedema patients who have either been misdiagnosed or who have received low attention in healthcare might face more challenges accessing healthcare services and in coping with their health situation.

This study also found that the period from lipoedema onset to actually receiving a lipoedema diagnosis by a healthcare professional could extend over decades, which aligns with a recently published study showing that the median time from lipoedema onset to diagnosis was 20–25 years [49]. This delay is problematic because it increases the risk of disease progression, which may cause even more negative health consequences. Recently published standards of care for lipoedema in the United States claim that a timely diagnosis is crucial in order to allow for early interventions and support that can reduce pain, maintain mobility, slow down the disease’s progression, and improve patients’ HRQOL [33]. The reason that lipoedema is so underdiagnosed has previously been explained by a limited knowledge on this disease within the wider healthcare field [50]. Moreover, the bodily appearance of lipoedema can often cause stigmatization (i.e. fat shaming) that then creates barriers for these women’s opportunities to be taken seriously by healthcare professionals [15]. However, guidelines that aim to improve care by enhancing recognition, diagnosis, and treatment for women diagnosed with it have increased in recent years [33, 35, 51, 52], as well as the fact that, recently, specific gene expressions in lipoedema stem cells have been identified, which also indicates possibilities for future diagnostic biomarkers [53].

Furthermore, this diagnosis delay may leave impacted women alone in terms of striving to understand and cope with their health situation, which, in turn, is facilitated by their inner health resources in SOC, including manageability, comprehensibility, and meaningfulness. Our results revealed that a stronger SOC was more associated with older age than with lipoedema characteristics. This result could be explained by the fact that SOC overall tends to increase with age [30, 54]. Women diagnosed with lipoedema later in life (i.e. age 60+ years) report a stronger SOC than do younger diagnosis age groups. However, conclusions made from these results should be drawn with caution because this group naturally includes women 60 years or older, meaning that the age factor might explain SOC. From this study’s findings, we can elucidate that, even though older age was found to be associated with a stronger SOC, it was also observed to be related to worsened physical health. This result should be understood from the perspective that even though a stronger SOC is associated with better health, it does not alone explain a person’s overall health status. The rest of the variance herein is explained or accounted for by other factors, such as age, social support, and education level [24]. Notably, there are no recommended exact values for defining low or high SOC scores, with SOC primarily not being suggested as a screening instrument or comparison item between groups. Instead, SOC outcomes must be interpreted in relation to a given context [22]. By conducting the, presumably, first study on SOC among women with lipoedema, we aimed to bring attention to and gain new knowledge on how SOC is distributed in a lipoedema population. Based on this, future research and healthcare interventions should focus on how a person’s inner health resources, including manageability, comprehensibility, and meaningfulness, can be promoted and supported when they are diagnosed with lipoedema.

This study does have some limitations. Due to non-existent national public authority registers or other Swedish lipoedema data sources, participants were exclusively recruited from Lipoedema Association groups, which may affect our findings’ external validity. As such, the proportion of confirmed lipoedema diagnoses among participants in this study is probably not representative of a general lipoedema population. There may also have been a selection bias because we have no information about those who did not participate, and that 1 in 4 of those who did participate were self-diagnosed. All data were self-reported, meaning that uncertainty may have occurred among participants in questions on lipoedema characteristics and retrospective questions on their health. Due to the challenges in recruiting participants with this considerably unknown disease, we did not reach the minimum sample size. Some of the subgroups were small, and therefore, cautions should be made in drawing general conclusions. Nevertheless, it should be noted that the mean differences for HRQOL, in most groups, were far larger than is proposed for a minimal clinical importance.

## Conclusions

This study found that having the loose connective tissue disease lipoedema is strongly associated with poorer physical health and a negative impact on HRQOL, which, in addition, tends to worsen as the disease progress into its more advanced stages. Despite chronic pain and substantial limitations in daily activities and social life due to the associated health problems, many women live with lipoedema symptoms and uncertainty regarding their health for decades before being correctly diagnosed by a healthcare professional. The results from this study strongly indicate a failure in early detection, which then suggests that many women are forced to cope with health problems with either no or deficient support from healthcare services. Moreover, this exploratory study on SOC among women with lipoedema brings attention to inner health-promoting resources in a lipoedema population that may be useful in future research on how women with lipoedema mobilise and utilise their internal and external health resources; more specifically, how these women manage their health problems and which experiences they have of healthcare.

## Supplementary Information

**Additional file 1.** Factor loadings for 41 scored symptoms in the lipoedema symptom severity questionnaire.

## Data Availability

The datasets generated and analyzed during this study are not publicly available due to ethical restrictions. For further information related to this dataset, please contact the corresponding author.

## Abbreviations

HRQOL: Health-related quality of life
SOC: Sense of coherence

## Footnotes

1 Refers to the total percentage among participants, separated from the factor analysis and therefore not presented in Fig. 2.

2 The item for measuring hypermobile joints was manually extracted in the factor analysis and was, therefore, not included in the nine-factor solution.
