# Supplementary material for: Women with lipoedema: a national survey on their health, health-related quality of life, and sense of coherence
Source: BMC Womens Health. 2022 Nov 18;22:457. doi: 10.1186/s12905-022-02022-3 (PMC9673372; doi:10.1186/s12905-022-02022-3)
Supplement: Supplementary file 1 — Additional file 1. Factor loadings for 41 scored symptoms in the lipoedema symptom severity questionnaire. [file 12905_2022_2022_MOESM1_ESM.docx]

**Additional file 1.** Factor loadings for 41 scored symptoms in the lipoedema symptom severity questionnaire.

| **Factor** | **Variable/Item** | **Factor scores** | | | | | | | | | |
| --- | --- | --- | --- | --- | --- | --- | --- | --- | --- | --- | --- |
| **Factor 1**  Pain in shoulders and neck | Shoulder pain while sitting | .792 |  |  |  |  |  |  |  |  |  |
|  | Shoulder pain while lying down | .775 |  |  |  |  |  |  |  |  |  |
|  | Shoulder pain during movement | .751 |  |  |  |  |  |  |  |  |  |
|  | Neck pain while sitting | .824 |  |  |  |  |  |  |  |  |  |
|  | Neck pain while lying down | .803 |  |  |  |  |  |  |  |  |  |
|  | Neck pain during movement | .766 |  |  |  |  |  |  |  |  |  |
| **Factor 2**  Pain in feet, leg, and skin | Foot pain while sitting |  | .763 |  |  |  |  |  |  |  |  |
|  | Foot pain while lying down |  | .810 |  |  |  |  |  |  |  |  |
|  | Foot pain during movement |  | .549 |  |  |  |  |  |  |  |  |
|  | Leg pain while sitting |  | .731 |  |  |  |  |  |  |  |  |
|  | Leg pain while lying down |  | .732 |  |  |  |  |  |  |  |  |
|  | Leg pain during movement |  | .518 |  |  |  |  |  |  |  |  |
|  | Skin pain during no touch situations |  | .602 |  |  |  |  |  |  |  |  |
|  | Skin pain during movement |  | .524 |  |  |  |  |  |  |  |  |
| **Factor 3**  Pain in buttocks and hip | Buttock pain while sitting |  |  | .536 |  |  |  |  |  |  |  |
|  | Buttock pain while lying down |  |  | .677 |  |  |  |  |  |  |  |
|  | Buttock pain during movement |  |  | .678 |  |  |  |  |  |  |  |
|  | Hip pain while sitting |  |  | .719 |  |  |  |  |  |  |  |
|  | Hip pain while lying down |  |  | .789 |  |  |  |  |  |  |  |
|  | Hip pain during movement |  |  | .762 |  |  |  |  |  |  |  |
| **Factor 4**  Leg heaviness,  swollenness, stiffness,  physical exhaustion | Leg heaviness |  |  |  | .724 |  |  |  |  |  |  |
|  | Leg swollenness |  |  |  | .726 |  |  |  |  |  |  |
|  | General swollenness |  |  |  | .690 |  |  |  |  |  |  |
|  | Joint stifness |  |  |  | .605 |  |  |  |  |  |  |
|  | Physical exhaustion |  |  |  | .640 |  |  |  |  |  |  |
|  | Aching muscles |  |  |  | .530 |  |  |  |  |  |  |
| **Factor 5**  Pain, heaviness, and numbness  in arms | Arm heaviness |  |  |  |  | .680 |  |  |  |  |  |
|  | Arm numbness |  |  |  |  | .571 |  |  |  |  |  |
|  | Arm pain while sitting |  |  |  |  | .709 |  |  |  |  |  |
|  | Arm pain while lying down |  |  |  |  | .699 |  |  |  |  |  |
|  | Arm pain during movement |  |  |  |  | .681 |  |  |  |  |  |
| **Factor 6**  Pain in back | Back pain while sitting |  |  |  |  |  | .834 |  |  |  |  |
|  | Back pain while lying down |  |  |  |  |  | .763 |  |  |  |  |
|  | Back pain during movement |  |  |  |  |  | .785 |  |  |  |  |
| **Factor 7**  Pain in hands | Pain in hands while at rest |  |  |  |  |  |  | .765 |  |  |  |
|  | Pain in hands during movement |  |  |  |  |  |  | .749 |  |  |  |
| **Factor 8**  Cold skin,  Feeling cold ,  bruising | Cold skin |  |  |  |  |  |  |  | .759 |  |  |
|  | Feeling cold |  |  |  |  |  |  |  | .840 |  |  |
|  | Easy bruising |  |  |  |  |  |  |  | .630 |  |  |
| **Factor 9**  Sleep problems | Sleep problems, physical-related |  |  |  |  |  |  |  |  | .655 |  |
|  | Sleep problems,  stress-related |  |  |  |  |  |  |  |  | .764 |  |
